# Radiomics and Machine Learning in Diagnostics of Glial Brain Tumors: a Systematic Review and Meta-Analysis

**DOI:** 10.17691/stm2025.17.6.07

**Published:** 2025-12-29

**Authors:** G.V. Danilov, S.B. Agrba, Yu.V. Strunina, A.M. Shevchenko, T.A. Konakova, S.V. Shugay, A.I. Batalov, I.N. Pronin

**Affiliations:** MD, PhD, Scientific Board Secretary, Head of the Laboratory of Biomedical Informatics and Artificial Intelligence; N.N. Burdenko National Medical Research Center for Neurosurgery, Ministry of Health of the Russian Federation, 16, 4th Tverskaya-Yamskaya St., Moscow, 125047, Russia; MD, PhD, Specialist, Laboratory of Biomedical Informatics and Artificial Intelligence; N.N. Burdenko National Medical Research Center for Neurosurgery, Ministry of Health of the Russian Federation, 16, 4th Tverskaya-Yamskaya St., Moscow, 125047, Russia; Leading Engineer, Laboratory of Biomedical Informatics and Artificial Intelligence; N.N. Burdenko National Medical Research Center for Neurosurgery, Ministry of Health of the Russian Federation, 16, 4th Tverskaya-Yamskaya St., Moscow, 125047, Russia; Radiologist, Department of X-ray and Radioisotope Diagnostic Techniques; N.N. Burdenko National Medical Research Center for Neurosurgery, Ministry of Health of the Russian Federation, 16, 4th Tverskaya-Yamskaya St., Moscow, 125047, Russia; Radiologist, Department of X-ray and Radioisotope Diagnostic Techniques; N.N. Burdenko National Medical Research Center for Neurosurgery, Ministry of Health of the Russian Federation, 16, 4th Tverskaya-Yamskaya St., Moscow, 125047, Russia; Pathologist, Department of Pathology; N.N. Burdenko National Medical Research Center for Neurosurgery, Ministry of Health of the Russian Federation, 16, 4th Tverskaya-Yamskaya St., Moscow, 125047, Russia; MD, PhD, Radiologist, Department of X-ray and Radioisotope Diagnostic Techniques; N.N. Burdenko National Medical Research Center for Neurosurgery, Ministry of Health of the Russian Federation, 16, 4th Tverskaya-Yamskaya St., Moscow, 125047, Russia; MD, DSc, Professor, Academician of the Russian Academy of Sciences, Deputy Director; N.N. Burdenko National Medical Research Center for Neurosurgery, Ministry of Health of the Russian Federation, 16, 4th Tverskaya-Yamskaya St., Moscow, 125047, Russia

**Keywords:** glial tumors, radiomics, machine learning, MR imaging of glial tumors, molecular biomarkers

## Abstract

Glial tumors are the most common neuroepithelial neoplasms of the brain. Consequently, investigating robust, non-invasive techniques for subtyping these tumors — specifically through advanced multimodal neuroimaging and radiomics — is warranted.

The present systematic review of scientific literature, including meta-analysis, was conducted to specify the major challenges of radiomics and machine learning in diagnostics of glial tumors based on the MRI data as well as to assess the quality of such non-invasive diagnostics.

We analyzed 42 publications utilizing radiomics and machine learning to predict molecular biomarker status in glial tumors based on MRI data. The analysis covered mutations in the *IDH*, *ATRX*, *BRAF*, and *H3K27M* genes, as well as *TERT* promoter mutations, 1p/19q codeletion, MGMT promoter methylation, and proliferative activity (Ki-67 labeling index). The overall accuracy of these techniques was high and equaled 0.86 [0.83; 0.89]. At the same time, the studies demonstrated significant methodological heterogeneity, in particular, related to the lack of uniform standards to select the location, size, and shape of the area of interest for obtaining radiomic features. This greatly hinders reproduction of the experimental results in clinical practice. Therefore, standardization of radiomics procedures remains relevant for further research of glial tumors.

## Introduction

Glial tumors (gliomas) are the most common neuroepithelial neoplasms of the brain [1]. In recent years, developments in molecular genetics resulted in a more sophisticated understanding of glioma genotypes, their biological heterogeneity, and biomarkers associated with treatment efficacy and patient survival [2]. Current standards for glioma diagnosis and treatment in most cases are based on tumor biopsy results, including molecular genetic analysis.

At the same time, the search for highly informative non-invasive techniques to subtype glial tumor (primarily with modern multimodal neuroimaging) remains relevant. Studies utilizing this technique are based on the hypothesis that imaging phenotypes reflect the histological and molecular characteristics of biological tissue. Hence, quantitative characteristics of shapes and textures in medical images can be considered as potential biomarkers. Computer science technologies used to search for radiological markers being correlates of biological characteristics of tissues are called “radiomics” and (in the context of predicting genetic nature of pathology) “radiogenomics” [3].

Modelling the complex relationship between a large number of imaging characteristics and tumor biological properties at present can be accomplished by using machine learning [4]. Machine learning is a means to generate mathematical models using large amounts of accumulated data. Mathematical models are “trained” to diagnose a tumor in a medical image using a sufficient number of clinical examples with the known diagnoses [5]. The most accessible and informative technique for the primary non-invasive diagnosis of gliomas is magnetic resonance imaging (MRI) in various modalities.

This systematic review of scientific literature followed by meta-analysis was conducted to identify the major challenges of using radiomics and machine learning in diagnosing gliomas with MRI data and to assess the quality of such non-invasive diagnostics.

## Materials and Methods

The study was conducted in accordance with the international PRISMA (Preferred Reporting Items for Systematic Reviews and Meta-Analyses) guidelines for systematic reviews and meta-analyses [6].

Scientific publications were selected for analysis should they comply with the following criteria:

1. the publication was indexed in the PubMed database;
2. the article was published in a scientific journal;
3. the article was written in English;
4. the type of article was original scientific research;
5. the study was dedicated to a quantitative MRI study of human glial brain tumors;
6. the publication calculated radiomics parameters based on MRI data;
7. the technical objective of the study was to classify MRI images;
8. machine learning algorithms (including deep learning) were used to classify MRI images;
9. the calculation of machine learning quality metrics was performed on a test sample;
10. the full text of the article was available.

Hence, the analysis did not include studies aimed at differential diagnostics of gliomas and other pathologies (metastases and other tumors of the nervous system, post-radiation pseudoprogression, inflammatory process, etc.), systematic reviews and meta-analyses of publication data, unpublished articles, and conference materials.

### Strategy to search literature

The literature search was performed using PubMed (https://pubmed.ncbi.nlm.nih.gov/) of the US National Library of Medicine (last accessed on August 20, 2023) with the following query: “featur* AND (deep learning OR DL OR machine learning OR ML) AND (biomark* OR histol* OR mutat* OR genotyp*) AND (stratif* OR classification OR grad* OR subtyp*) AND (radiomic*[Title/Abstract] OR radiogenomic*[Title/ Abstract]) AND (gliom* OR glial) AND (MRI OR magnetic resonance)”.

**Figure 1.**
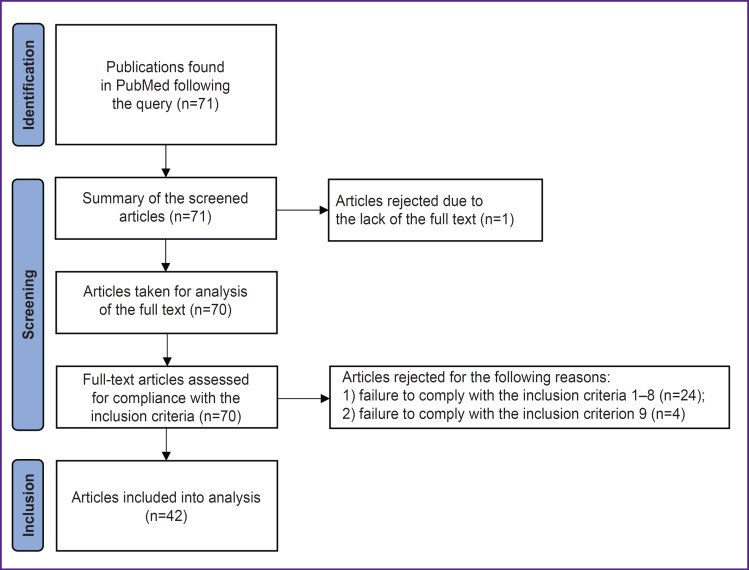
Scheme showing the process of selecting articles for the systematic review and meta-analysis according to the international PRISMA guidelines

This search resulted with 71 articles in English, with full-text access to 70 of them. A primary screening of the found publications for inclusion criteria was independently performed by one of the authors hereof. Another author independently performed a control screening (see the Author contributions section). Disagreements between the authors on inclusion of articles in the analysis were resolved by consensus. A diagram showing the selection results is demonstrated in Figure 1.

Forty-two articles [7–48] that fully met the inclusion criteria were selected for analysis.

### Statistical data analysis

Descriptive statistics and meta-analysis methods were used in this study. The distribution of continuous random variables was characterized by the median, 25%, and 75% quantiles [Q1; Q3]. Categorical variables are presented as percentages; 95% confidence intervals (95% CI) were calculated for the estimated values.

Meta-analysis procedures were used to obtain a generalized estimate of the accuracy, sensitivity, and specificity of machine learning models across the entire publication sample and its subsets. To standardize calculations, the authors took the numbers of true-positive, true-negative, false-positive, and false-negative predictions in the test samples from each publication (either explicitly or by calculations based on the data from the article). The statistical generalization results of the mentioned metrics were presented using a forest plot. Sample heterogeneity was assessed using the I^2^ metric. Due to the relatively small values of the studied effects, an intermediate logit transformation and a generalized linear mixed-effects model were used to calculate the generalized effect size.

Data analysis was performed using the R statistical programming language (version 4.2.1) in the RStudio Server IDE. The tidyverse, meta, metafor, and dmetar packages were utilized for the analysis.

## Results

The selected studies focused on developing machine learning models to predict various target variables using radiomics data. The target variables in the reviewed studies included molecular biomarkers: mutation status of the *IDH* (n=23), *ATRX* (n=4), *TERT* (n=3), and *BRAF* (n=1) genes, *H3K27M* (n=2), *EGFR* amplification (n=1), *MGMT* promoter methylation (n=3), Ki-67 expression (n=1), glioma WHO grade (n=2), 1p/19q codeletion (n=6), and several other characteristics of the glioma molecular profile. A wide range of radiation biomarkers was used as predictors, including first-order features, as well as quantitative indicators calculated using gray level co-occurrence matrices (GLCM), gray level run length matrices (GLRLM), neighbouring gray tone difference matrices (NGTDM), gray level size zone matrices (GLSZM), and gray level dependence matrices (GLDM).

The analyzed studies also contained information on sample size, glioma WHO grade, software to calculate radiological biomarkers, MRI field strength, applied MRI scan sequences, analyzed image size (2D/3D), segmentation techniques, training and test sample sizes, used machine learning models, and machine learning models with the highest performance metrics on test samples. The performance of machine learning models using radiomics data in the reviewed studies was assessed with the metrics of accuracy, sensitivity, specificity, F-score, positive and negative predictive value, and area under the ROC curve.

The sample size presented in the publications varied from 40 to 1508 MR studies (155 [102; 258]). In 25 (59.5%) studies, MR scanning was performed in T1, T1 with contrast enhancement, T2 and T2-FLAIR sequences, and additional modes were used in some series. In 16 series (38.1%), contrast-enhanced images were not analyzed. In 37 (88%) studies, calculations of radiation biomarkers were performed using volumetric (3D) areas of interest, of which 3 studies additionally studied flat (2D) areas. At that, in 5 studies, information on the dimension of the area of interest was not provided. The most frequently used software for feature extraction was ITK-SNAP (mentioned in 14 articles), segmentation technology based on the expectation maximization (EM) algorithm being the basis for the glioma image segmentation and registration (GLISTR) technique (mentioned in 5 publications), and 3D-Slicer (mentioned in 4 articles). Eight articles did not provide information on software for segmentation areas of interest. In 20 studies (47.6%), the calculation of radiological biomarkers was performed using the PyRadiomics library, in 5 papers MatLab was used, and in 10 publications information on software for calculation radiological biomarkers was not provided.

**Figure 2.**
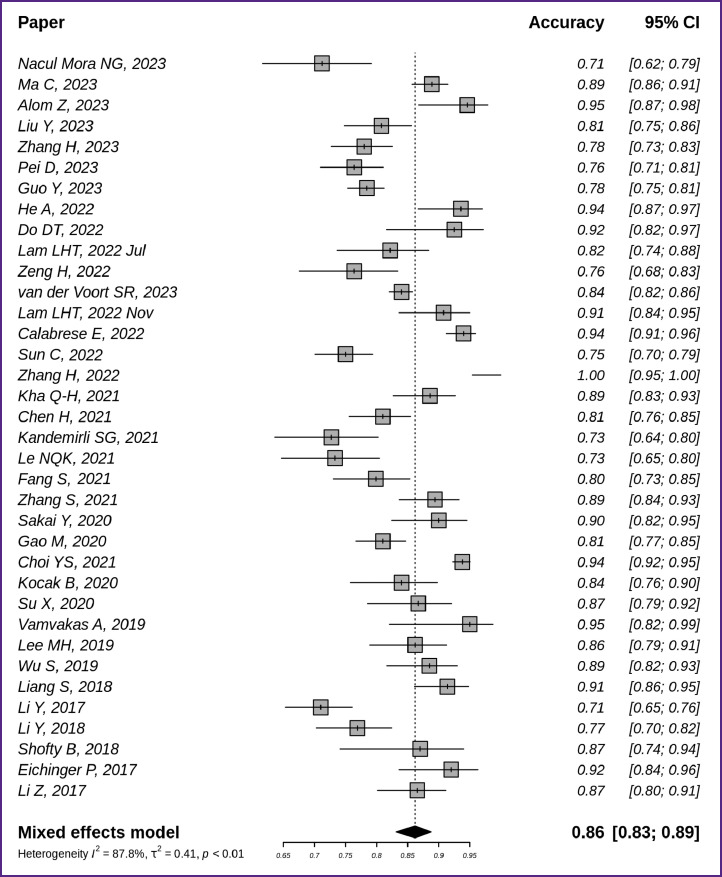
Accuracy of glioma MR image classification techniques based on radiomics and machine learning (n=36)

Figure 2 [7–42] demonstrates the accuracy of machine learning models and its generalization for 36 of 42 papers for which the authors managed to assess accuracy metrics. The generalized accuracy in the analyzed publications was 0.86 with 95% CI [0.83; 0.89].

**Figure 3.**
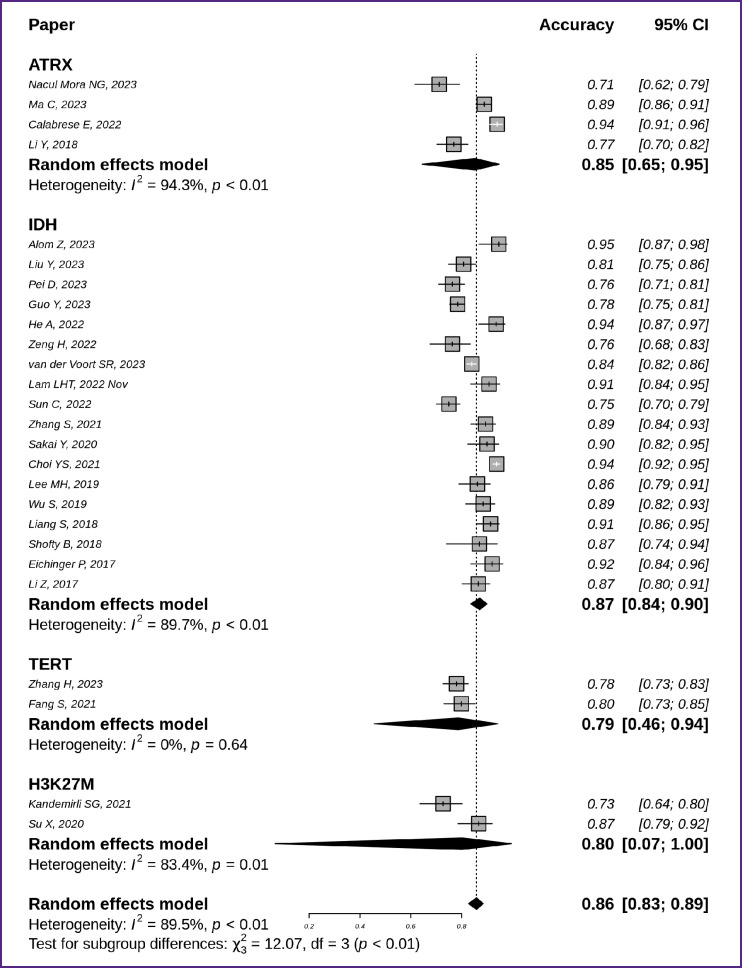
Accuracy of techniques to identify molecular markers — *IDH* (n=18), *ATRX* (n=4), *TERT* (n=2), and *H3K27M* (n=2) — in glioma MR images

The accuracy of classification models in prediction of specific molecular markers and the generalization of this accuracy across series of studies are shown in Figure 3 [7–14, 17–21, 25, 27–29, 31, 33, 35–37, 39–42]. The highest generalized accuracy was demonstrated by models to predict the *IDH* mutation status at 0.87 with a 95% CI [0.84; 0.90] and the *ATRX* mutation status at 0.85 with a 95% CI [0.65; 0.95]. Here, the *TERT* and *H3K27M* prediction models showed lower accuracy values and wider confidence intervals (0.79 with 95% CI [0.46; 0.94] and 0.80 with 95% CI [0.07; 1.00], respectively).

**Figure 4.**
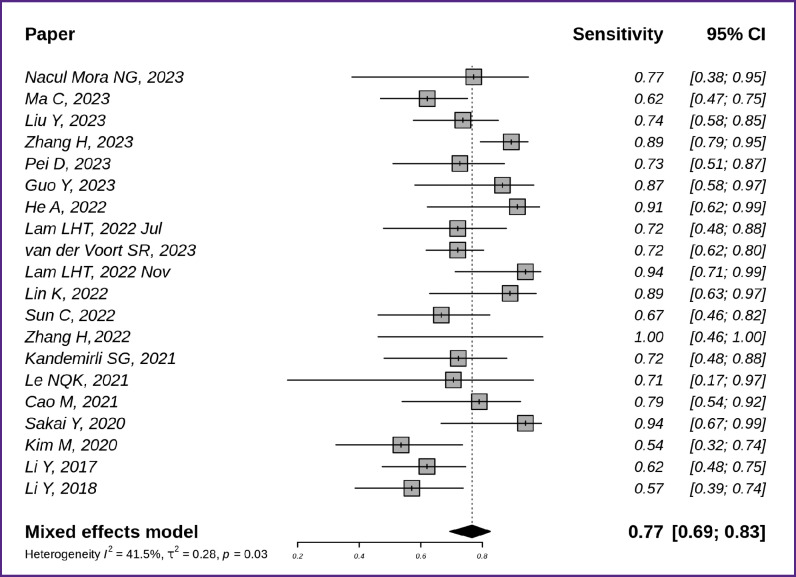
Sensitivity of glioma MR image classification techniques based on radiomics and machine learning (n=20)

Figure 4 [7, 8, 10–14, 16, 18, 19, 21, 22, 25, 26, 29, 38, 39, 43–45] demonstrates the sensitivity of non-invasive glioma subtyping techniques based on radiomics and machine learning in the 20 studies reviewed. The generalized sensitivity in the reviewed publication series was 0.77 with a 95% CI [0.69; 0.83].

**Figure 5.**
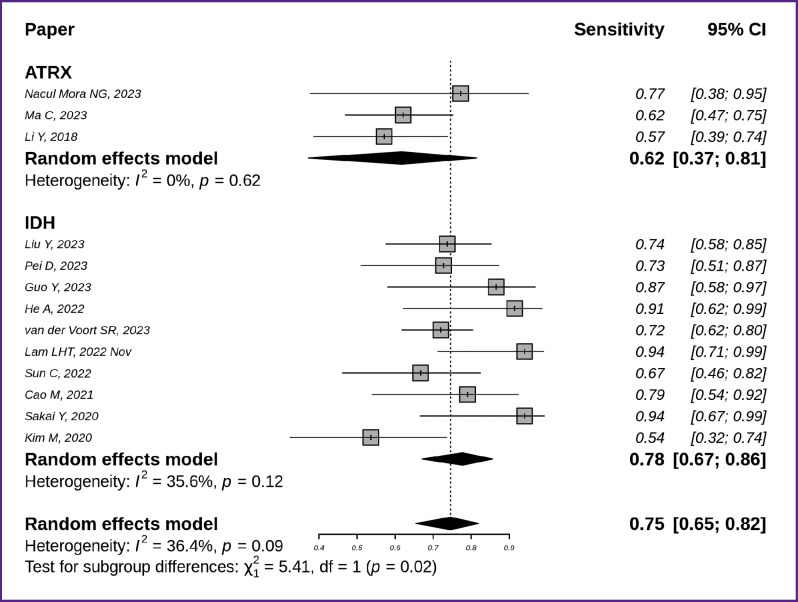
Sensitivity of the techniques to identify the *IDH* (n=10) and *ATRX* (n=3) molecular markers in glioma MR images

The sensitivity of the techniques for detecting the *IDH* and *ATRX* gene mutation status is shown in Figure 5 [7, 8, 10, 12–14, 18, 19, 21, 29, 39, 44, 45]. The generalized sensitivity of the models for *IDH* mutation detection equalled to 0.78 with a 95% CI [0.67; 0.86], while for *ATRX* this metric was lower — 0.62 with a wider 95% CI [0.37; 0.81].

**Figure 6.**
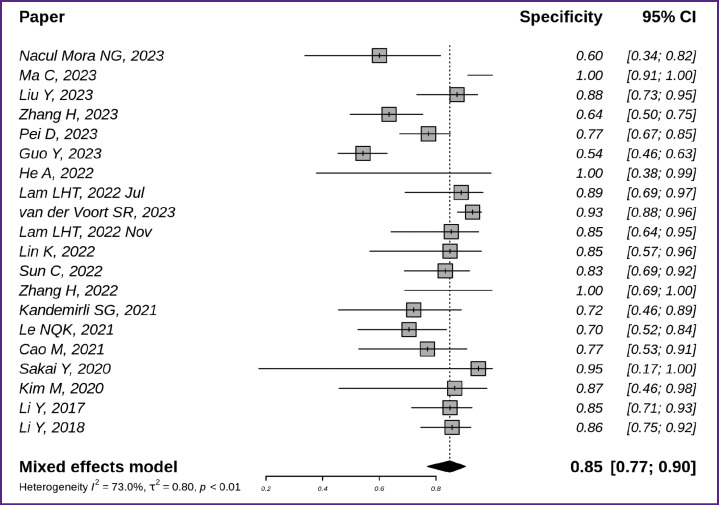
Specificity of glioma MR image classification techniques based on radiomics and machine learning (n=20)

**Figure 7.**
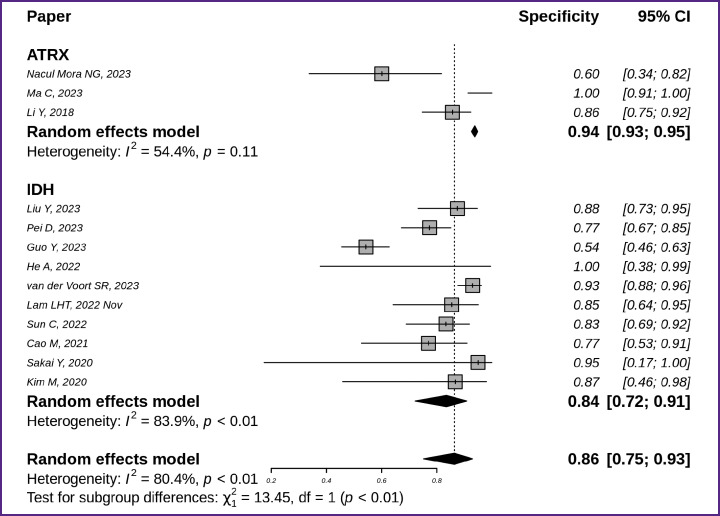
Specificity of techniques to identify *IDH* (n=10) and *ATRX* (n=3) molecular markers in glioma MR images for specific target variables

The specificity of diagnostics based on radiomics and machine learning is demonstrated in Figures 6 and 7. The overall specificity of the studied techniques exceeded the sensitivity and was equal to 0.85 with a 95% CI [0.77; 0.90] (Figure 6) [7, 8, 10–14, 16, 18, 19, 21, 22, 25, 26, 29, 38, 39, 43, 44, 45].

The highest specificity was highlighted for detecting *ATRX* mutations (0.94 with 95% CI [0.93; 0.95]); it was slightly lower for identifying *IDH* mutations (0.84 with 95% CI [0.72; 0.91]) (Figure 7) [7, 8, 10, 12–14, 18, 19, 21, 29, 39, 44, 45].

## Discussion

The conducted literature review revealed fairly high-quality metrics of machine learning models in non-invasive glial tumor subtyping. Here, the analyzed studies demonstrated significant methodological variety in choosing target variables, MRI sequences, software tools for segmentation, calculating MR biomarkers, and other technical aspects.

The authors believe that, paradoxically, the principle of specifying areas of interest by delineating the visible tumor signal, which is repeated in all studies, brings significant methodological heterogeneity into the process of calculating MR biomarkers. The emerging heterogeneity in results is explained by two reasons: 1) the discrepancy between the visible boundaries of gliomas (e.g., areas of contrast enhancement) and the real presence of tumor cells in the brain; 2) the arbitrary and variable sizes and shapes of the areas of interest within which MR biomarkers are calculated. Given that the absolute values of radiomic features can depend on the size of the area of interest, this dependency compromises the reliability of such measurements [49].

The authors believe that a critical limitation of current glial tumor radiomics studies is the lack of assessment regarding the reproducibility and robustness of the extracted biomarkers and machine learning models.

Heterogeneity in the reviewed studies was seen not only in the research process but also in the quality and completeness of the results. For instance, a number of studies lacked references to fundamental aspects of image analysis technique, such as the selection of the area of interest and its type, the software used, the field strength of the MR scanner, etc. The Image Biomarker Standardization Initiative (IBSI), an international association working on standardization of processes for obtaining radiological biomarkers for quantitative analysis, proposed recommendations that, if followed, would minimize methodological heterogeneity and increase the results reproducibility [49]. References to these recommendations were seen in some publications in the analyzed series [17, 24].

Another limitation of studies on glial tumor radiomics is the relatively small sample size (rarely exceeding 300 studies). Considering the current variability in this tumor imaging and the diversity of its genetic variants, such a sample size appears to be insufficient to fully detect various digital patterns as well as to test the reproducibility of radiological biomarkers. The development of biobanking tools integrating various clinical, laboratory, and instrumental data, as well as the accumulation of a significant data base (including from multicenter studies) will likely allow to overcome this shortcoming in the future.

A little over half of all the reviewed studies were related to predicting the *IDH* gene mutation status (see Figures 3, 5, 7). In some recent studies authors proposed approaches to automated glioma segmentation (based on deep machine learning) and getting radiological biomarkers [17, 18, 46]. Here, neural network models used so-called deep features to extract specific digital image features. Such deep features were obtained not by using predetermined formulas, but as an intermediate product of the neural network’s machine learning process. Some studies demonstrated that combining “classical” radiomic features with deep features improved the accuracy of tumor genetic variant prediction [20].

The current classification of nervous system tumors is based on the results of molecular and genetic studies, which are crucial for prescription of the most effective targeted treatment [1]. In the era of genetic technologies development, the knowledge of the tumor nature is expanding. At that, access to expensive and labor-intensive genetic testing in hospitals is limited. Therefore, the search for non-invasive correlates of genetic and molecular biomarkers in nervous system tumors, including radiological correlates, appears to be a promising research area. However, despite the success of radiomics-based research, there is still much to be done to assess such technologies reliability, reproducibility, and suitability for use in daily clinical practice.

## Conclusion

Research into radiomics and machine learning has yielded promising results for the non-invasive diagnosis of glial tumors. At the same time, issues of reliability and reproducibility of the proposed solutions remain relevant. It is advisable to follow IBSI recommendations when using radiomics technologies to analyze MR images of glial tumors and when publishing the results thereof.
